# Mahimbrine A, a Novel Isoquinoline Alkaloid Bearing a Benzotropolone Moiety from *Mahonia imbricata*

**DOI:** 10.3390/molecules23071539

**Published:** 2018-06-26

**Authors:** Mao-Sheng Zhang, Yan Deng, Shao-Bin Fu, Da-Le Guo, Shi-Ji Xiao

**Affiliations:** 1 School of Pharmacy, Zunyi Medical University, Zunyi 563006, China; maosheng.zhang@163.com (M.-S.Z.); 13658521539@163.com (Y.D.); fushb@126.com (S.-B.F.); 2 School of Pharmacy, Chengdu University of Traditional Chinese Medicine, Chengdu 611137, China; guodale2008@163.com

**Keywords:** *Mahonia imbricata*, Berberidaceae, isoquinoline alkaloid, mahimbrine A

## Abstract

A novel isoquinoline alkaloid, mahimbrine A, possessing a rare benzotropolone framing scaffold, was isolated from the endemic plant of *Mahonia imbricata*. Its structure was established on the basis of extensive spectroscopic analysis. A plausible biosynthetic route of mahimbrine A was proposed. Mahimbrine A showed no antimicrobial activity at the concentration of 1 mg/mL.

## 1. Introduction

**Figure 1:**
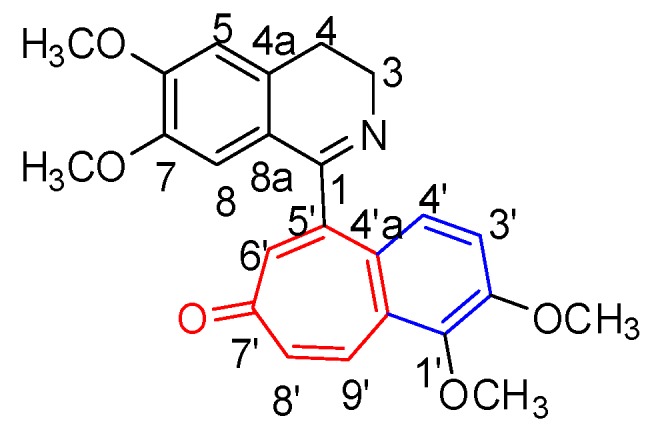
Structure of mahimbrine A.

*Mahonia imbricata* Ying *et* Boufford, as one of the endemics of seed plants in China, is a perennial shrub of the family Berberidaceae, distributed only in Guizhou and Yunan provinces of Southwest China [1]. Plants of the genus *Mahonia* have long been used as a traditional medicine to treat tuberculosis, periodontitis, dysentery, pharyngolaryngitis, eczema, and wounds [2]. Previous chemical investigations on this genus have involved a series of chemical constituents. Among these constituents, alkaloids are principal constituents of the genus *Mahonia* [3,4,5]. As part of an ongoing research program to isolate and determine structures of secondary metabolites from medicinal endemic plants of southwestern China [6,7], we performed a phytochemical study on *M. imbricate.* As a result, a novel isoquinoline alkaloid, possessing a rare benzotropolone substituent, was isolated (Figure 1). Its structure was established by means of spectroscopic analysis including one- and two-dimensional NMR spectroscopy. Moreover, the hypothetical biosynthetic route was proposed. Mahimbrine A was tested against four gram-positive bacterial strains and four gram-negative bacteria.

## 2. Results and Discussion

### 2.1. Structure Elucidation of Mahimbrine A

Mahimbrine A was obtained as a brown gum. Its molecular formula was established as C_24_H_23_NO_5_ on the basis of the HR-ESI-MS ([M + H]^+^ at *m*/*z* 406.1632, calcd. 406.1649 and [2M + H]^+^ at *m*/*z* 811.3226, calcd. 811.3225), which requires 14 degrees of unsaturation. The IR absorptions at 3434 and 1633 cm^−1^ suggested the presence of hydroxyl and carbonyl groups in the molecule. UV absorptions at λ_max_ 230 and 288 nm deduced the presence of an α, β-unsaturated carbonyl moiety [8]. The ^1^H, ^13^C NMR and HSQC spectra of mahimbrine A (Table 1) showed 24 carbon resonances due to a methine at δ_H_ 2.91 (2H, m), δ_C_ 25.8; four methoxyl groups at δ_H_ 3.60 (3H, s), 3.93 (6H, s), 3.94 (3H, s), δ_C_ 56.2, 56.2, 56.2, 61.8; a methine at δ_H_ 3.90 (1H, m), 4.03 (1H, m), δ_C_ 47.9; a 1,2,3,4-tetrasubstituted phenyl ring moiety at δ_H_ 7.04 (1H, d, *J* = 9.1 Hz), 7.28 (1H, d, *J* = 9.1 Hz), δ_C_ 153.7, 148.4, 1131.5, 129.2, 128.8, 114.5; a 1,2,4,5-tetrasubstituted phenyl ring moiety at δ_H_ 6.57 (1H, s), 6.78 (1H, s), δ_C_ 151.8, 147.9, 131.4, 121.9, 110.6, 110.6; a carbonyl at δ_C_ 188.4; and an unsaturated system at δ_H_ 8.20 (1H, d, *J* = 13.0 Hz), 6.87 (1H, dd, *J* = 13.0, 2.7 Hz), 6.74 (1H, d, *J* = 2.7 Hz), δ_C_ 168.4, 148.8, 134.9, 133.6, 133.5.

**Figure 2:**
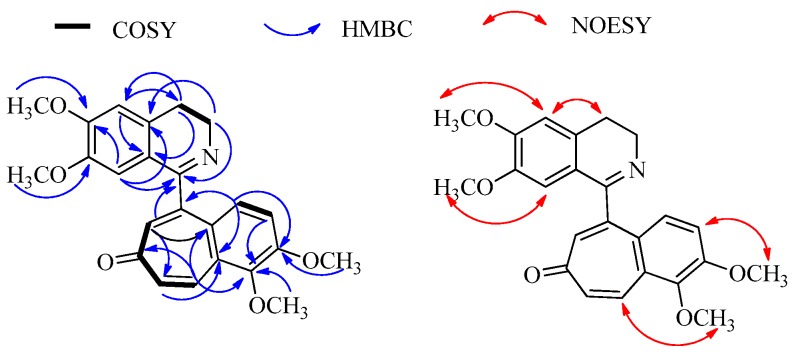
Key COSY, HMBC and NOESY correlations of mahimbrine A.

The obvious HMBC correlations (Figure 2) from H-3 to C-1, C-4a, H-4 to C-5, C-4a, C-8a, H-5 to C-4, C-7, C-8a, H-8 to C-1, C-4a, C-6, OCH_3_ to C-6 and OCH_3_ to C-7 evidenced the presence of a dihydroisoquinoline unit with two methoxyl groups located at C-6 and C-7, respectively. The HMBC correlations of H-9′ to C-7′ (δ_C_ 188.4), C-4′a, H-8′ to C-6′, C-9′a (δ_C_ 131.5), and H-6′ to C-4′a (δ_C_ 129.2), C-8′a, established the presence of a tropolone moiety, which was further verified by the vicinal coupling constants of H-8′/H-9′ (*J* = 13.0 Hz) [9]. Moreover, the HMBC correlations of H-9′ to C-1′, C-4′a, H-4′ to C-2′, C-5′, C-9′a confirmed the tropolone moiety and 1,2,3,4-tetrasubstituted phenyl ring moiety connected via a bridged bond at C-4′a and C-9′a. Similarly, two methoxyl groups at C-1′ and C-2′ were also assigned. The HMBC correlations of H-6′ to C-1, C-4′a and H-4′ to C-5 indicated that the dihydroisoquinoline unit and benzotropolone unit were connected to each other by C-1 and C-5′. Thus, the final structure of this compound was determined and named mahimbrine A.

### 2.2. Plausible Biogenetic Pathway

**Scheme 1:**
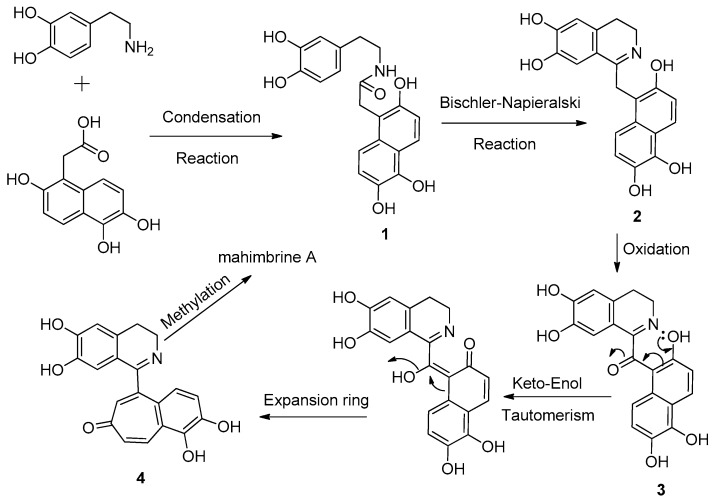
Proposed Biogenetic Pathway of mahimbrine A.

A plausible biogenetic pathway for mahimbrine A was postulated (Scheme 1). As a precursor, naphthylacetic acid and dopamine via condensation reaction to give an amide intermediate **1** [10], which subsequently undergoes a Bischler-Napieralski reaction to generate intermediate **2** [11]. Intermediate **2** is then oxidized to yield intermediate **3** [12]. Intermediate **3** can spontaneously convert to its enol form. This enol intermediate then undergoes ring expansion rearrangement to give intermediate **4** [13], which is finally methylated to get mahimbrine A.

### 2.3. Antimicrobial Activity

The antimicrobial activity of mahimbrine A was evaluated against four gram-positive bacterial strains *Staphylococcus aureus* ATCC 6538, *Micrococcus luteus* CMCC 28001, *Staphylococcus epidermidis* ATCC 12228, and *Bacillus subtilis* ATCC 21332; and four gram-negative bacteria *Pseudomonas aeruginosa* ATCC 27853, *Escherichia coli* ATCC 25922, *Salmonella typhimurium* ATCC 14028, and *Enterobacter aerogenes* ATCC 13048, by a microdilution titre technique; neither was active [14]. Gentamicin and streptomycin were used as positive controls. Discs containing 10 μL DMSO solutions were used as a negative control. All tests were performed in triplicate.

## 3. Experimental Section

### 3.1. General Procedures

UV spectra were recorded on a Perkin-Elmer Lambda 35 UV-VIS spectrophotometer (Perkin-Elmer, Waltham, MA, USA). IR spectra were measured on a PerkinElmer one FT-IR spectrometer (KBr) (Perkin-Elmer, Waltham, MA, USA). 1D and 2D-NMR spectra were recorded on a Bruker-Ascend-400 or an Agilent DD2400-MR instrument using TMS as the internal reference. HR-ESI-MS spectra were measured on a LTQ Orbitrap XL mass spectrometer (Thermo Scientific, Waltham, MA, USA). Column chromatography was performed using silica gel (300–400 mesh, Qingdao marine Chemical Ltd., Qingdao, China). Semi-preparative HPLC was performed on LC3000 system (Beijing ChuangXingTongHeng Science And Technology Co., Ltd., Beijing, China) equipped with an ODS column (5 μm, i.d. 10 mm × 250 mm, YMC). Original raw data of 1D, 2D NMR spectra and HR-ESI-MS of mahimbrine A are available in the supplementary materials.

### 3.2. Plant Material

The plant was collected from Loushanguan in Zunyi City, People’s Republic of China, in October 2013, and identified as *Mahonia imbricata* by Prof. Jian-Wen Yang, Zunyi Medical University. A voucher specimen (20131020) was deposited with the Herbarium of the School of Pharmacy, Zunyi Medical University.

### 3.3. Extraction and Isolation

The air-dried and finely ground stems of *Mahonia imbricata* (30 kg) were extracted with 90% ethanol for three times at room temperature (5 days each). The ethanol extract was evaporated under reduced pressure to get a crude extract (2 kg), which was further suspended in acidic water (pH 3–4) and then filtered to obtain the filtrate. The filtrate was alkalized to pH 9–10 and extracted successively with CHCl_3_ and n-BuOH (each 3 times), respectively. The CHCl_3_ extract was subjected to silica gel column chromatography (80 mm × 600 mm, 1400 g, 300–400 mesh), eluted with a gradient of CHCl_3_/MeOH (*v*/*v* = 100:1–1:100) to afford ten fractions (A–J). Fraction C was separated by Sephadex LH-20 column chromatography (CHCl_3_/MeOH = 1:1) and divided into 15 subfractionses (fraction A.1–A.15). Mahimbrine A (3.8 mg) was obtained from fraction A.6 by sempipreparative HPLC (MeOH/H_2_O = 55:45, flow rate 4 mL/min).

### 3.4. Spectral Data

Mahimbrine A: brown gum; UV (CH_3_OH): 230 (2.68), 288 (2.43); IR (KBr): 3434, 2920, 1633, 1590, 1512, 1384, 1292, 1026, 810; ^1^H and ^13^C NMR data, Table 1. HR-ESI-MS: *m*/*z* 406.1632 [M + H]^+^, C_24_H_24_NO_5_^+^, calcd. 406.1649; *m*/*z* 811.3226 [*2*M + H]^+^, calcd. 811.3225.

## 4. Conclusions

In conclusion, mahimbrine A has been isolated from the endemic plant of *Mahonia imbricate* and its chemical structure has been elucidated. It displays an interesting structure with a dihydroisoquinoline moiety bound to a rare benzotropolone ring system. The structure of mahimbrine A was elucidated by HR-ESI-MS, 1D, 2D NMR and a biosynthetic pathway was proposed. Mahimbrine A did not show antimicrobial activity at the tested concentration of 1 mg/mL. The interesting structural architecture of this natural product might find further applications.

## Figures and Tables

**Table 1:** ^1^H and ^13^C NMR data for mahimbrine A (400/100 MHz, in CDCl_3_).

Position	δ_H_	δ_C_	Position	δ_H_	δ_C_
1		168.4	1′		148.4
3	4.03, 3.90, m	47.9	2′		153.7
4	2.91, m	25.8	3′	7.04, d (9.1)	114.5
4a		131.4	4′	7.28, d (9.1)	128.8
5	6.78, s	110.6	4′a		129.2
6		151.8	5′		148.8
7		147.9	6′	6.74, d (2.7)	133.5
8	6.57, s	110.6	7′		188.4
8a		121.9	8′	6.87, dd (13.0, 2.7)	134.9
6-OCH_3_	3.94, s	56.2	9′	8.20, d (13.0)	133.6
7-OCH_3_	3.60, s	56.2	9′a		131.5
1′-OCH_3_	3.93, s	61.8			
2′-OCH_3_	3.93, s	56.2			

## Supplementary Materials

1D, 2D NMR spectra and HR-ESI-MS of mahimbrine A are available online.
