# Supplementary figures and images for: Mahimbrine A, a Novel Isoquinoline Alkaloid Bearing a Benzotropolone Moiety from Mahonia imbricata
Source: Molecules. 2018 Jun 26;23(7):1539. doi: 10.3390/molecules23071539 (PMC6100064; doi:10.3390/molecules23071539)

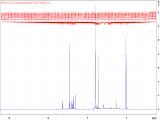

Supplement: Supplementary file 1 [file molecules-23-01539-s001.zip › NMR/1H NMR CDCl3/1/pdata/1/thumb.png]

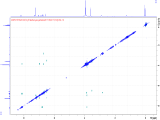

Supplement: Supplementary file 1 [file molecules-23-01539-s001.zip › NMR/NOESY CDCl3/1/pdata/1/thumb.png]
